# Study of Microvessel Density and the Expression of Vascular Endothelial Growth Factors in Adrenal Gland Pheochromocytomas

**DOI:** 10.1155/2014/104129

**Published:** 2014-09-03

**Authors:** Magdalena Białas, Grzegorz Dyduch, Joanna Dudała, Monika Bereza-Buziak, Alicja Hubalewska-Dydejczyk, Andrzej Budzyński, Krzysztof Okoń

**Affiliations:** ^1^Department of Pathomorphology, Jagiellonian University Medical College (UJCM), Grzegórzecka Street 16, 31-531 Cracow, Poland; ^2^Faculty of Physics and Applied Computer Science, AGH-University of Science and Technology, Aleja Mickiewicza 30, 30-059 Cracow, Poland; ^3^Jagiellonian University Medical College, Kopernika Street 17, 31-501 Cracow, Poland; ^4^II Department of General Surgery, Jagiellonian University Medical College, University Hospital, Kopernika 21 Street, 31-501 Cracow, Poland

## Abstract

Angiogenesis (neoangiogenesis), a process of neovascularization, is an essential step for local tumor growth and distant metastasis formation. We have analysed angiogenesis status: vascular architecture, microvessel density, and vascular endothelial growth factors expression in 62 adrenal pheochromocytomas: 57 benign and 5 malignant. Immunohistochemical evaluation revealed that vascular architecture and vessel density are different in the central and subcapsular areas of the tumor. Furthermore, we have observed a strong correlation between number of macrophages and microvessel density in the central and subcapsular areas of the tumor and between the expression of VEGF-A in tumor cells and microvessel density in central and subcapsular areas of the tumor. Secondary changes in these tumors influence the results and both vascular architecture and microvessel density are markedly disturbed by hemorrhagic and cystic changes in pheochromocytomas. These changes are partially caused by laparoscopic operation technique. However, no differences in vascular parameters were found between pheochromocytomas with benign and malignant clinical behavior. Our observation showed that analysis of angiogenesis, as a single feature, does not help in differentiating malignant and benign pheochromocytomas and has no independent prognostic significance. On the other hand, high microvessel density in pheochromocytoma is a promising factor for antiangiogenic therapy in malignant cases.

## 1. Introduction

Adrenal pheochromocytoma (PCC) is an uncommon, neuroendocrine, catecholamine-secreting tumour arising from chromaffin cells of the adrenal medulla [1, 2]. Its clinical behavior is uncertain [3–5]. The histological separation between benign and malignant cases is usually difficult, and a definitive diagnosis of malignant PCC should be restricted to lesions displaying distant metastases [1, 2]. Metastases are defined by the finding of tumour cells in sites where chromaffin cells are normally absent [6]. According to most authors, recurrent disease occurring months or even years after the initial operation allows for classification of the tumour to a malignant group [1, 6, 7]. The most common metastatic sites are lymph nodes, liver, lungs, and bones [1, 2, 8]. PASS criteria (Pheochromocytoma of the Adrenal Gland Scaled Score) were proposed in 2002 as a tool for differentiating between benign and malignant cases [9] but this scoring system is not perfect and has some limitations. Using this scale, a significant proportion of PCC receives boundary PASS values (PASS = 4 or 5) which do not allow for an unequivocal assignment of the tumor to a benign or malignant group. It is necessary to find additional features that allow better prediction of the clinical malignant behavior of the tumor (future recurrence or metastasis). Microvessel density (MVD) may be such parameter.

Neoangiogenesis, a process of neovascularization, is a complex phenomenon which plays a vital role in many physiological processes like organ development, wound healing, and tissue regeneration as well as in the pathology of many diseases, especially inflammatory and neoplastic diseases [10]. Angiogenesis is also essential for tumor growth and metastasis formation. Cancers, after a so-called angiogenic switch, acquire the ability to induce new vessel formation. The process of neovascularization depends on the ability to release specific factors stimulating and inhibiting new blood vessel formation. Both blood vessels formation stimulating and inhibiting factors can be released by neoplastic cells, stromal components, and immune cells like macrophages. Many strategies are used to evaluate the role of neoangiogenesis in tumor progression, and one of them is assessing microvessel density (MVD) [11, 12].

The aim of the study was to compare MVD, expression of vascular endothelial growth factors (VEGF-A, VEGF-C, and VEGF-D), and the number of macrophages in different areas of 57 benign and 5 malignant tumors and to determine if angiogenesis evaluation can be useful in routine pathomorphological practice for predicting the clinical outcome of a particular PCC tumor.

## 2. Material and Methods

The PCC samples were obtained from the Pathomorphology Department, Medical College Jagiellonian University (MCJU) in Krakow, Poland. The study was approved by the Jagiellonian University Bioethical Committee (KBET/82/B/2010).

The material under study consisted of 62 PCCs diagnosed in 58 patients (30 males and 28 females) in the Pathomorphology Department of MCJU during a period of 15 years from 1996 to 2010. Four patients, three women and one man, had bilateral tumours. Seven patients were known to have one of the syndromes associated with increased incidence of adrenal pheochromocytoma (two patients with MEN 2A syndrome, four with NF, and one with VHL syndrome). Three of these patients (two with MEN 2A and one with NF syndrome) had bilateral tumours. Ten patients were 30 or younger—only one tumor in this group presented malignant clinical behaviour. The mean tumour diameter was 4.98 cm (median: 4.2 cm, range: 1.5–13 cm, SD = 2.38) with no significant difference between male and female group (5.1 and 4.9 cm, resp.)—Table 1.

Five tumours were malignant: three PCCs gave distant metastases (to liver, lungs, and bones) and two had locally recurred. All PCCs with a malignant clinical course were unilateral. Clinical data were derived from patients' records and were available in 49 of the 58 cases (mean time of the follow-up: 46.3 months, median: 39 months). Nine cases were lost to follow-up, but we know that these patients were not treated for any recurrence and/or metastasis in our department.

Haematoxylin and eosin-stained slides and paraffin blocks from tumors and adjacent adrenal glands were available in all 62 cases. Each diagnosis of PCC was reevaluated and confirmed by immunohistochemical staining with four antibodies against chromogranin A (CrA), synaptophysin (Syn), S-100 protein (S-100), and melan A [13]. Tumour cell immunoreactivity for chromogranin and synaptophysin with simultaneous lack of immunoreactivity for melan A and the presence of S-100 positive elongated cells, at least focally, was taken as confirmation of the diagnosis of PCC. Severity of haemorrhagic changes in the tumor was estimated in each case. The hemorrhagic changes within the tumor were scored from 0 (none or minimal) to 3 (extensive hemorrhagic changes disrupting at least half of the tumor surface visible in the histological slides).

In each case, a single H-E section and corresponding paraffin block including well-preserved tumor tissue as well as capsule were chosen, and seven 3 *μ*m sections were prepared from the paraffin block. MVD was evaluated after immunostaining endothelial cells with antibodies against CD31 and CD105 for blood vessels and D2-40 for lymphatic vessels. Additionally, the expression of vascular endothelial growth factors (VEGF-A, VEGF-C, and VEGF-D) was evaluated. Immunohistochemistry was performed by standard method: the slides were dewaxed, rehydrated, and incubated in 3% peroxide solution for 10 minutes to block the endogenous peroxidase activity. Antigen retrieval was carried out by microwaving in citrate buffer (pH 6.0) or EDTA for 5 minutes at 700 W and then for 5 minutes at 600 W. The Lab-Vision (Thermo Fisher Scientific, Waltham, USA) detection system was used. 3-Amino-9-ethylcarbazole served as the chromogen. The slides were counterstained with Mayer's haematoxylin (DAKO, Denmark). The primary antibodies and the respective technical details are summarised in Table 2.

Positive structures (Figures 3 and 4), morphologically identifiable vessels and collections of immunopositive cells as well as single endothelial cells, were counted independently by two of the authors (MB and GD) who were blinded to the clinical and pathological data in two different areas of the tumor: the subcapsular and intratumoral spaces of each tumor. The subcapsular space was defined as the area within one high power field (0.5 mm) beneath the outer border of the tumor. The remainder of the tumor was defined as the intratumoral (central) area.

Two different methods of counting were used. In the first method the number of all vessels in 10 high power fields, HPF (×10 ocular, ×40 objective), was added up after prescanning on low magnification (×10 ocular, ×10 objective) to choose the area with the impression of the highest vessel profiles number (“hot spot”) in the intratumoral space. In the subcapsular area all vessels in 10 consecutive HPF were counted. The result calculated was the mean count of vessels for one HPF. In the second method, the Chalkley eyepiece graticule (Chalkley grid area 0.196 mm^2^) with 25 randomly positioned dots was applied to the ocular of the Olympus microscope. On higher magnification (×10 ocular, ×40 objective) a Chalkley eyepiece graticule was applied to each “hot spot” area and then orientated and rotated so that the maximum number of points would hit on or within the vessel structure in the “hot spot” area. In the Chalkley method dots are counted, not the individual vessels. The Chalkley count was expressed as the total number of dots per square millimeter.

Lymphatic vessels (after D2-40 immunostaining (Figure 5)) were counted subcapsularly in 10 consecutive HPF and in 10 HPF in the intratumoral space.

Macrophages were counted in 10 HPF after prescanning on low magnification (×10 ocular, ×10 objective) to choose the area with higher cell density. The result calculated was the mean count of CD68 positive cells for one HPF.

The extent of immunoreactivity for VEGFs was expressed as the sum of grade and intensity of staining. Staining was graded according to the percentage of positive tumour cells (0: no staining; 1: <10%; 2: 10–50%; 3: 51–100% of positive cells). Intensity of staining was described as none (0), weak (1), moderate (2), or strong (3) (Figures 6–9). As a result, combined VEGF immunoreactivity could range from 0 to 6. All evaluations were done using an Olympus BX51 microscope equipped with a 40x UPlanFLN eyepiece (field of view diameter: 0.55 mm).

Statistical analysis was performed using Statistica 10 (StatSoft Inc., Tulsa, USA). Comparison between groups was done with Student's *t*-test, Mann-Whitney *U* test, and Kruskall-Wallis ANOVA test; the relationship between variables was assessed using Spearman's correlation coefficient. The significance level was set to 0.05.

## 3. Results

The material consisted of 62 cases of PCC from 58 patients: 30 males and 28 females. Three females and one man had bilateral PCC. The average age of the patients was 47.66 years (range: 19 to 75, SD: 15.41). The age in males and females (48.42 versus 46.90) did not differ significantly. 29 tumors (46.8%) were located at the right adrenal gland, 21 tumors (33.9%) were located at the left adrenal gland, and in 4 patients tumors were bilateral. In 4 cases (6.4%) laterality was not stated.

Angiogenesis was evaluated by MVD by two different methods. The overall results showing the number of blood vessels in the subcapsular and central areas of tumors are summarized in Table 3.

**Figure 1:**
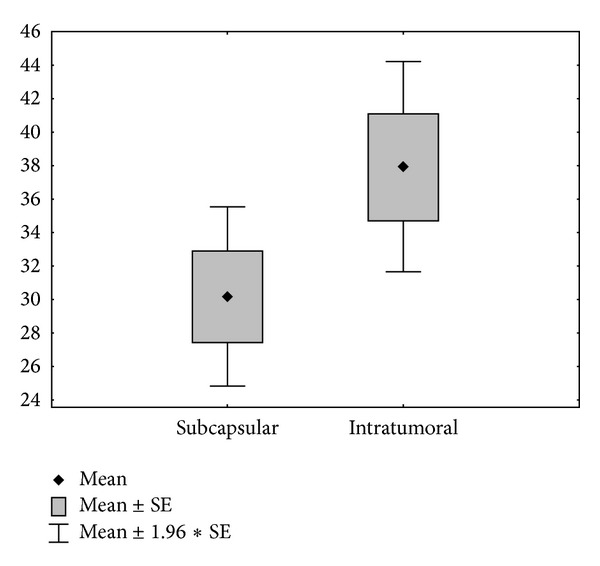
Number of CD105 positive blood vessels in subcapsular and central areas of the tumors.

In both counting methods, MVD in the central areas of the tumors was higher than in the subcapsular areas. Strong correlation was found between both the numbers of CD31 positive blood vessels in subcapsular and central areas of the tumors (*r* = 0.8653, *P* < 0.001) and between the numbers of CD105 positive blood vessels in subcapsular and central areas of the tumors (*r* = 0.8837, *P* < 0.01)—Figure 1. The difference between the variables for CD105 positive vessels was statistically significant (30.155 versus 37.91, Student's *t*-test *P* < 0.001).

Lymphatic vessels were absent in central parts of all investigated PCCs. In 4 cases (6,4%) single lymphatic D2-40 positive vessels were present in subcapsular areas. In 55 PCCs few lymphatic vessels were present within the capsule.

**Figure 2:**
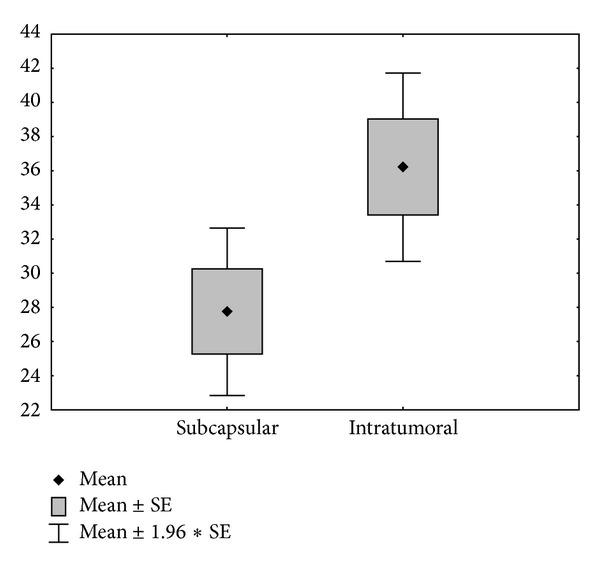
Number of macrophages in subcapsular and central areas of the tumors.

**Figure 3:**
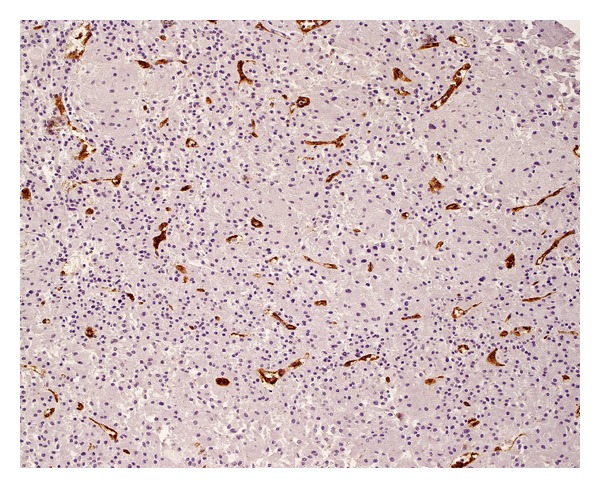
Numerous blood vessels with small round lumens (immunostaining for CD31).

**Figure 4:**
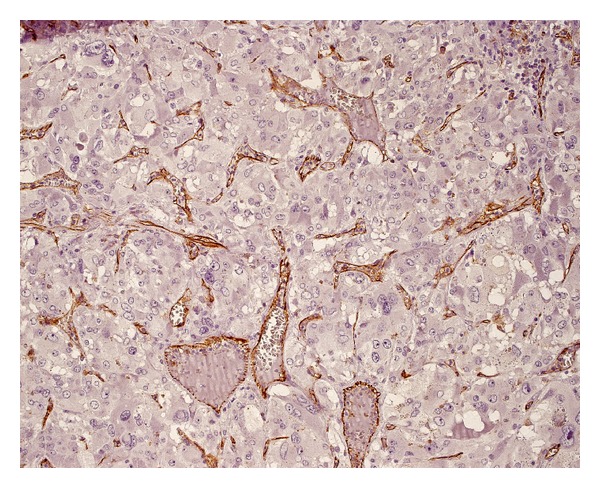
Blood vessels with irregular, expanded lumens (immunostaining for CD31).

**Figure 5:**
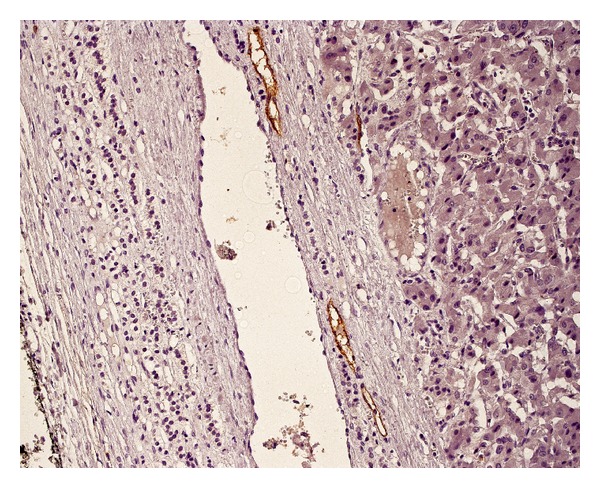
Lymphatic blood vessels present only in the tumor capsule (immunostaining for D2-40).

**Figure 6:**
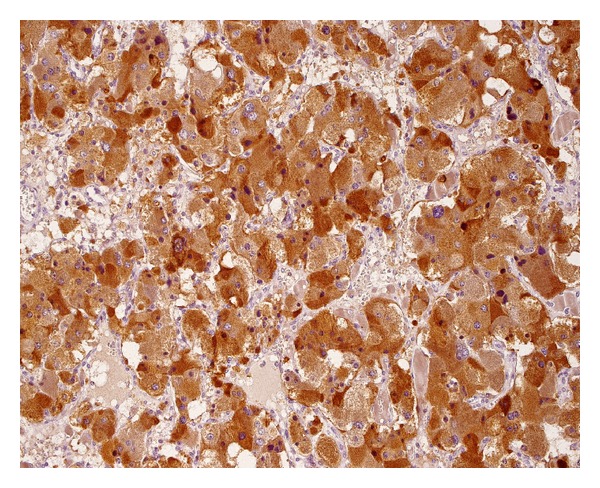
Positive, strong (3+/2+), granular immunostaining for VEGF-A in 100% of tumor cells.

**Figure 7:**
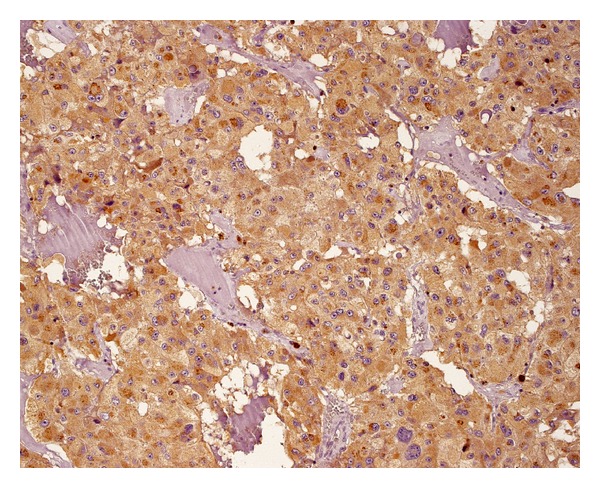
Positive (2+) immunostaining for VEGF-A in about 10% of tumor cells.

**Figure 8:**
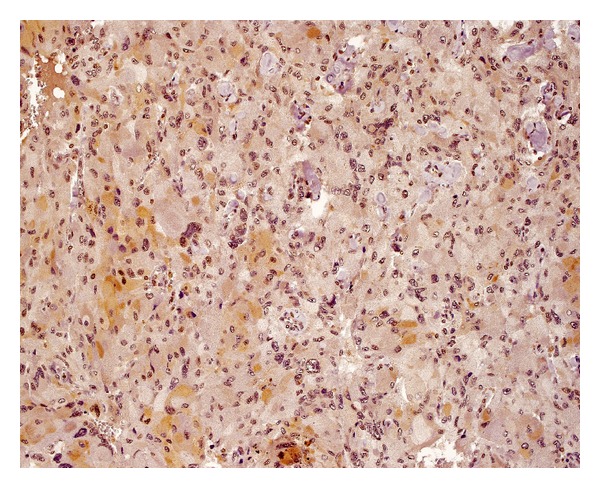
Immunostaining for VEGF-C weakly positive (1+) in 20% of tumor cells.

**Figure 9:**
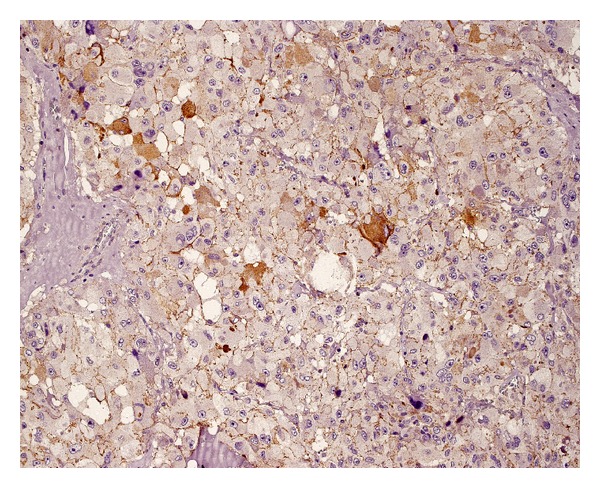
Immunostaining for VEGF-D only focally positive (2+) in single cells.

Mean subcapsular CD68 positive cell count was 27.68 (range: 4 to 87, SD: 18.97); mean intratumoral CD68 positive cell count was 36.14 (range: 11 to 97, SD: 21.24)—Figure 2. Strong correlation was found between the numbers of macrophages in subcapsular and central parts of the tumors (*r* = 0.9166, *P* < 0.01) and the difference between the values (27,68 versus 36,14) was statistically significant (Wilcoxon and Student's *t*-test *P* < 0.001).

Strong correlation was found between the number of macrophages and the number of CD31 positive blood vessels and between the number of macrophages and CD105 positive blood vessels in subcapsular and central parts of the tumors—all correlation coefficients were statistically significant (*P* < 0.0001)—Table 4.

The detailed individual values and statistical analysis for VEGFs expression are presented in Table 5. VEGF-A showed the strongest expression and was correlated with the number of both intratumoral and subcapsular CD31 positive and CD105 positive vessels in both counting methods—Table 6. No correlation was found between the expression of VEGF-C and MVD and between VEGF-D expression and MVD.

An inverse correlation between haemorrhagic changes and the number of CD105 positive vessels in subcapsular parts of the tumor was found (*P* = 0.018).

The differences in vascular parameters between PCCs with benign and malignant clinical behavior were slight and not statistically significant—Table 7.

## 4. Discussion

Angiogenesis (neoangiogenesis, NA) is the formation of new capillaries from already existing vessels. NA is regulated by a variety of proteins, inter alia, vascular growth factors and their receptors, angiogenesis modulating proteins, integrins, and angiogenesis inhibitors [14–16]. NA is a complex phenomenon and many strategies are used to evaluate its role in physiological and pathological processes. Most commonly used methods consist of assessing microvessel density (MVD) and expression of angiogenic factors, among which the most important are vascular endothelial growth factors (VEGFs) and their receptors (VEGFRs) [11, 12, 17]. NA is essential both in nonpathological processes like embryogenesis or wound healing and in tumorigenesis [10, 18] where it is an essential step for tumor growth, progression, and metastasis formation [11]. Formation of new blood capillaries is also dependent on the extracellular matrix which serves as structural support for existing and developing vessels and on the ability of different cells to release specific factors stimulating new blood vessel formation and factors which downregulate vessel formation inhibitors [19]. The sources of those factors are both neoplastic cells and various stromal and immune cells, inter alia, macrophages. Microvascular density can be a prognostic factor in some human cancers [17, 20, 21], as metastasis formation is dependent on the possibility of tumor cells to enter the lumen of small vessels and to flow with blood to distant places and organs. Importantly, this means that neovascularisation is necessary not only for local tumor growth but also for allowing distant spread of the neoplasm.

The currently accepted standard method for quantifying tumor angiogenesis is to assess MVD based on immunohistochemistry (IHC). Groups of scientists had chosen different antibodies to evaluate MVD in various tumors [17, 22–26]. Our group had found in previous studies that the choice of IHC marker used for endothelial cells detection may influence the results, and the CD31 antibody as an endothelial marker provides the most unequivocal and conspicuous results [27]. CD31-highlighted endothelial cells are clearly visible and easy to count. On the other hand, CD34 antibody highlighted not only blood vessels but also other structures in the vicinity, such as connective tissue fibers, and usually CD34 gives much higher counts than CD31 [26]. Another endothelial marker commonly used in assessing MVD is endoglin (CD105). CD105 is a proliferation-associated and hypoxia-inducible protein abundantly expressed in angiogenic endothelial cells. Endoglin is a receptor for transforming growth factor- (TGF-) beta1 and TGF-beta3, and it modulates TGF-beta signalling. CD105 is required for endothelial cell proliferation [28], and CD105-based MVD is an independent prognostic factor for survival in patients with some tumor types [29–31]. CD105 is strongly expressed in the blood vessels of tumor tissues.

We have investigated the angiogenic status by comparing vascular architecture, microvessel count (based on both CD31 and CD105 IHC), and the expression of VEGFs in different areas of benign and malignant PCC tumors.

In the analyzed group of PPC, both benign and malignant neoplasms were highly vascularized tumors. Vascular architecture pattern was not equal, and vascular channels had different shapes and sizes in different parts of the tumor. Favier et al. had reported the differences in vascular architecture between benign and malignant PPC: benign tumors exhibited a regular pattern of small vessels while malignant PCC exhibited a more irregular pattern of vessels along with the presence of larger vascular channels between tumor cell nodules [24]. We have found highly heterogeneous vascular architecture patterns within particular PCC tumors, both benign and malignant. In areas with hemorrhagic and/or cystic changes, relevant quantification of vascular pattern was much more difficult and results were not always reliable. Changing operating techniques (prevailing laparoscopic procedures) increases the incidence and extent of hemorrhages in adrenal tumors (data prepared for publication) and therefore assessment of vascular architecture seems not to be a reliable procedure in PCC.

Angiogenesis (NA) was evaluated by assessing MVD using immunohistochemistry with CD31 and CD105 and assessing the expression of VEGFs. We have found that the MVD was higher in central areas of the tumor compared with subcapsular areas for both vessel counting strategies and with the use of both antibodies (CD31 and CD105). A strong correlation was found between the numbers of CD31 and CD105 positive blood vessels in both subcapsular and central areas of the tumors. The difference between the variables for CD105 positive vessels was statistically significant (30.155 versus 37,91, Student's *t*-test *P* < 0.001). This could be an indication that NA is more efficient in oxygen-reduced central parts of the tumor. Low oxygen conditions activate the hypoxia signaling pathway in neoplastic cells. Hypoxia-inducible target genes mediate multiple biological functions involved in the development of new blood vessels. Oxygen deprivation shifts the balance between factors stimulating and inhibiting angiogenesis toward the former.

We have also observed a strong correlation between the number of macrophages (in both subcapsular and central areas of the tumors) and MVD assessed by IHC with both CD31 and CD105 and between expression of VEGF-A in the tumor cells and MVD. A more than twofold excess in VEGF-A expression level was observed compared to VEGF-D levels. Expression of VEGF-A was also higher than expression of VEGF-C. The overexpression of VEGF-A and correlation between the number of macrophages and MVD indicate that neoangiogenesis in PCC is VEGF-A dependent and macrophages are highly involved in the process. VEGF-C and VEGF-D seem to be less involved in the vascularization of PCC. As we have stated in a previous study, mast cells also participate in vessel formation in PCC [32].

There are reports that MVD could influence the prognosis of various solid tumors. The literature concerning angiogenic status in PCC is still scanty and the results are ambiguous; some authors had found an increase in vascular density (MVD) in malignant versus benign PCC but some did not confirm these results [23, 24, 33–39]. Our investigation showed that there was no correlation between angiogenic status of PCCs and their malignant (recurrent or metastatic) behavior. We did not observe overexpression of any VEGFs or higher MVD in malignant versus benign PCCs, but the lack of significant differences in MVD and VEGF expression between groups of PCC in our study may be due to a small number of cases in the second investigated group. Increase in MVD in malignant PCCs was previously described by Favier in a group of PCCs, 50% of which harbour the SDHB-mutation (so-called cluster 1 tumors, C1) and were mostly extraadrenal PCCs (paragangliomas). The group of tumors analysed in our study consisted of 62 adrenal PPCs in which SDH-mutations are very rare—only two of 62 tumors (3,2%) harbour SDHB-gene mutations (data prepared for publication). On the other hand, Ohij et al. reported the absence of statistical association between MVD and malignancy in PCC [38].

Lymphatic vessel density was analyzed in the same 62 PCC tumors after IHC with the lymphatic endothelial marker D2-40. D2-40 labelling revealed a complete absence of lymphatic vessels in the central parts of all PCCs. We have found single lymphatic vessels in 4 PCCs (6,4%) in subcapsular areas and in 55 PCCs (88.7%) within the capsule. With only a few lymphatic vessels that are found only in the subcapsular areas of the tumor, it can be assumed that the spread through lymphatics to lymph nodes will be much rarer than the spread by blood to distant organs.

For the majority of patients with both benign and malignant PCC, the surgical removal of the tumor is the treatment of choice. In malignant cases with distant metastases, chemotherapy (CVD combination: cyclophosphamide, vincristine, and dacarbazine), radiotherapy, and/or radiometabolic therapy using 131J-MIBG can also be used [39, 40]. These therapies may lead to remission and symptom relief in up to 50% of patients [22, 40]. Even so, half of the patients with malignant, metastatic PCC do not benefit from these therapies, and there is a need to find other treatment possibilities. Because all PCCs are highly vascularized neoplasms, malignant tumors may be candidates for molecular targeted therapies, especially antiangiogenic therapies targeting the vascular endothelial growth factor pathway. Monoclonal anti-VEGF antibody (bevacizumab) and tyrosine kinase inhibitors are already used in patients with advanced renal carcinoma and gastrointestinal stromal tumors (GIST).

In summary, PCCs differed in vascular density in central and subcapsular areas of the tumor, but there were no statistically significant differences in vascular density between benign and malignant cases, so MVD is not appropriate to differentiate between benign and malignant PPC. Moreover, secondary changes in these tumors influence the results and both vascular architecture and MVD are markedly disturbed by hemorrhagic and cystic changes in PCCs. These changes are partially caused by laparoscopic operation technique. High MVD in all PCCs is a promising factor for antiangiogenic therapy, especially in the subgroup of PCC belonging to the cluster 1 group (with SDHX or VHL-gene mutation [22].

## 5. Conclusion

1. Microvessel density, as a single feature, does not help in differentiating malignant and benign PCC and has no independent prognostic significance in PCC.
2. The results of assessing vascular architecture and MVD are biased by secondary changes in tumor tissue, especially hemorrhages and cystic changes.
3. High MVD in all PCCs is a promising factor for antiangiogenic therapy, especially in the subgroup of malignant PCC belonging to the cluster 1 group (with SDHX or VHL-gene mutation).

## Figures and Tables

**Table 1:** Characteristics of patients with the diagnosis of pheochromocytoma.

	Male	Female	Total
Age (y)	48.4	46.9	47.6
Site			
Left	11	17	24
Right	17	13	34
Unknown	3	1	4
Diameter (cm)	5.1	4.9	5.0
PASS	4.09	4.25	4.17

**Table 2:** Primary antibodies used in the study.

Specificity	Clone	Manufacturer	Dilution	Antigen retrieval
CD31	JC70A	DAKO, Denmark	1 : 20	EDTA
CD105	4G11	Novocastra	1 : 50	Citrate buffer
D2-40	D2-40	Covance	Ready to use	Citrate buffer
VEGF-A	Polyclonal	Santa Cruz	1 : 100	EDTA
VEGF-C	Polyclonal	Santa Cruz	1 : 100	—
VEGF-D	78923	R&D Systems	1 : 200	EDTA
CD68	PG-M1	DAKO, Denmark	1 : 50	EDTA

**Table 3:** The vessel counts in the whole study group of PCCs.

Marker	Location	Method	Mean	Min.	Max.	SD
CD31	Subcapsular	Hot spot	56.88	15.00	120.00	23.72
	Intratumoral	Hot spot	60.07	19.00	142.00	27.91
	Subcapsular	Chalkey	40.31	0.00	75.68	13.54
	Intratumoral	Chalkey	46.08	0.00	79.08	14.37

CD105	Subcapsular	Hot spot	30.15	6.00	120.00	20.84
	Intratumoral	Hot spot	37.91	9.00	124.00	24.48
	Subcapsular	Chalkey	23.51	0.00	70.58	12.75
	Intratumoral	Chalkey	33.15	0.00	64.63	15.22

D2-40	Capsular	Hot spot	1.92	0.00	7.00	1.34
	Subcapsular	Hot spot	0.12	0.00	4.00	0.56
	Intratumoral	Hot spot	0.00	0.00	0.00	—

**Table 4:** Correlation between the number of macrophages and the number of CD31 and CD105 positive blood vessels in subcapsular and central areas of the tumors.

CD68	CD31		CD105	
	Subcapsular	Intratumoral	Subcapsular	Intratumoral
Subcapsular	*r* = 0.4534	*r* = 0.4803	*r* = 0.6013	*r* = 0.5701
Intratumoral	*r* = 0.4972	*r* = 0.5391	*r* = 0.5806	*r* = 0.6124

**Table 5:** The expression of VEGF-A, VEGF-C, and VEGF-D.

	Mean	Min.	Max.	SD
VEGF-A	4.57	2	6	1.05
VEGF-C	3.58	0	6	1.09
VEGF-D	1.93	0	4	1.42

**Table 6:** Correlations between the expressions of VEGF-A and MVD (“hot spot” method).

	CD31		CD105	
	Subcapsular	Intratumoral	Subcapsular	Intratumoral
VEGF-C	*r* = 0.1757	*r* = 0.0637	*r* = 0.0999	*r* = −0.0227
VEGF-A	*r* = 0.3330	*r* = 0.4028	*r* = 0.4702	*r* = 0.4282
VEGF-D	*r* = −0.1068	*r* = −0.1526	*r* = −0.0193	*r* = −0.1338

**Table 7:** Vascular parameters in benign and malignant pheochromocytomas.

Marker	Location	Method	Benign		Malignant	
			Mean	SD	Mean	SD
CD31	Subcapsular	Hot spot	57.47	23.69	50.40	25.78
	Intratumoral	Hot spot	61.19	27.71	48.00	30.32
	Subcapsular	Chalkey	39.71	13.49	47.11	13.69
	Intratumoral	Chalkey	45.61	14.22	51.53	16.70

CD105	Subcapsular	Hot spot	29.94	21.07	28.60	20.26
	Intratumoral	Hot spot	37.75	23.61	39.60	35.91
	Subcapsular	Chalkey	23.09	13.13	28.23	5.69
	Intratumoral	Chalkey	32.34	15.01	42.35	16.22

D2-40	Capsular	Hot spot	1.87	1.32	2.50	1.73
	Subcapsular	Hot spot	0.11	0.57	0.20	0.45
	Intratumoral	Hot spot	0.00	0.00	0.00	0.00

VEGF-A			4.64	1.01	3.80	1.30

VEGF-C			3.60	1.12	3.40	0.89

VEGF-D			1.98	1.41	1.40	1.67

## References

[1] Miettinen M, Miettinen M (2010). Paragangiomas. *Modern Soft Tissue Pathology—Tumors and Non-Neoplastic Conditions*.

[2] Lack EE (2007). Tumors of the adrenal glands and extraadrenal paraganglia. *AFIP Atlas of Tumor Pathology*.

[3] McNicol AM (2011). Update on tumours of the adrenal cortex, phaeochromocytoma and extra-adrenal paraganglioma. *Histopathology*.

[4] de Wailly P, Oragano L, Radé F (2012). Malignant pheochromocytoma: new malignancy criteria. *Langenbeck's Archives of Surgery*.

[5] Agarwal A, Mehrotra PK, Jain M (2010). Size of the tumor and pheochromocytoma of the adrenal gland scaled score (PASS): can they predict malignancy?. *World Journal of Surgery*.

[6] DeLellis RA, Lloyd RV, Heitz PU, Eng C (2004). *Tumours of Endocrine Organs—Pathology and Genetics*.

[7] Plouin P-F, Chatellier G, Fofol I, Corvol P (1997). Tumor recurrence and hypertension persistence after successful pheochromocytoma operation. *Hypertension*.

[8] Pacak K, Eisenhofer G, Ahlman H (2007). Pheochromocytoma: Recommendations for clinical practice from the First International Symposium. *Nature Clinical Practice Endocrinology and Metabolism*.

[9] Thompson LDR (2002). Pheochromocytoma of the adrenal gland scaled score (PASS) to separate benign from malignant neoplasms: a clinicopathologic and immunophenotypic study of 100 cases. *The American Journal of Surgical Pathology*.

[10] Kumar V, Abbas AK, Fausto N (2010). *Robbins and Cotran Pathologic Basis of Disease*.

[11] Sharma S, Sharma MC, Sarkar C (2005). Morphology of angiogenesis in human cancer: a conceptual overview, histoprognostic perspective and significance of neoangiogenesis. *Histopathology*.

[12] Vermeulen PB, Gasparini G, Fox SB (2002). Second international consensus on the methodology and criteria of evaluation of angiogenesis quantification in solid human tumours. *European Journal of Cancer*.

[13] Białas M, Okoń K, Dyduch G (2013). Neuroendocrine markers and sustentacular cell count in benign and malignant pheochromocytomas—a comparative study. *Polish Journal of Pathology*.

[14] Bergers G, Benjamin LE (2003). Tumorigenesis and the angiogenic switch. *Nature Reviews Cancer*.

[15] Ferrara N (2002). VEGF and the quest for tumour angiogenesis factors. *Nature Reviews Cancer*.

[16] Nyberg P, Xie L, Kalluri R (2005). Endogenous inhibitors of angiogenesis. *Cancer Research*.

[17] Pallares J, Rojo F, Iriarte J, Morote J, Armadans LI, de Torres I (2006). Study of microvessel density and the expression of the angiogenic factors VEGF, bFGF and the receptors Flt-1 and FLK-1 in benign, premalignant and malignant prostate tissues. *Histology and Histopathology*.

[18] Carmeliet P, Jain RK (2000). Angiogenesis in cancer and other diseases. *Nature*.

[19] Fukumura D, Duda DG, Munn LL, Jain RK (2010). Tumor microvasculature and microenvironment: novel insights through intravital imaging in pre-clinical models. *Microcirculation*.

[20] Weidner N (1995). Intratumor microvessel density as a prognostic factor in cancer. *American Journal of Pathology*.

[21] Hasan J, Byers R, Jayson GC (2002). Intra-tumoural microvessel density in human solid tumours. *British Journal of Cancer*.

[22] Favier J, Igaz P, Burnichon N (2012). Rationale for anti-angiogenic therapy in pheochromocytoma and paraganglioma. *Endocrine Pathology*.

[23] Liu Q, Djuricin G, Staren ED (1996). Tumor angiogenesis in pheochromocytomas and paragangliomas. *Surgery*.

[24] Favier J, Plouin P-F, Corvol P, Gasc J-M (2002). Angiogenesis and vascular architecture in pheochromocytomas: distinctive traits in malignant tumors. *The American Journal of Pathology*.

[25] Trojan L, Thomas D, Friedrich D (2004). Expression of different vascular endothelial markers in prostate cancer and BPH tissue: an immunohistochemical and clinical evaluation. *Anticancer Research*.

[26] El-Gohary YM, Silverman JF, Olson PR (2007). Endoglin (CD105) and vascular endothelial growth factor as prognostic markers in prostatic adenocarcinoma. *American Journal of Clinical Pathology*.

[27] Białas M, Okoń K, Czopek J (2003). Assessing microvessel density in gastric carcinoma: a comparison of three markers. *Polish Journal of Pathology*.

[28] Zhang Y, Yang Y, Hong H, Cai W (2011). Multimodality molecular imaging of CD105 (Endoglin) expression. *International Journal of Clinical and Experimental Medicine*.

[29] Dallas NA, Samuel S, Xia L (2008). Endoglin (CD105): a marker of tumor vasculature and potential target for therapy. *Clinical Cancer Research*.

[30] Fonsatti E, Sigalotti L, Arslan P, Altomonte M, Maio M (2003). Emerging role of endoglin (CD105) as a marker of angiogenesis with clinical potential in human malignancies. *Current Cancer Drug Targets*.

[31] Rubatt JM, Darcy KM, Hutson A (2009). Independent prognostic relevance of microvessel density in advanced epithelial ovarian cancer and associations between CD31, CD105, p53 status, and angiogenic marker expression: a Gynecologic Oncology Group study. *Gynecologic Oncology*.

[32] Białas M, Dyduch G, Szpor J, Demczuk S, Okoń K (2012). Microvascular density and mast cells in benign and malignant pheochromocytomas. *Polish Journal of Pathology*.

[33] Rooijens PPGM, De Krijger RR, Bonjer HJ (2004). The significance of angiogenesis in malignant pheochromocytomas. *Endocrine Pathology*.

[34] Zielke A, Middeke M, Hoffmann S (2002). VEGF-mediated angiogenesis of human pheochromocytomas is associated to malignancy and inhibited by anti-VEGF antibodies in experimental tumors. *Surgery*.

[35] Feng F, Zhu Y, Wang X (2011). Predictive factors for malignant pheochromocytoma: analysis of 136 patients. *Journal of Urology*.

[36] Kolomecki K, Stepien H, Bartos M, Kuzdak K (2001). Usefulness of VEGF, MMP-2, MMP-3 and TIMP-2 serum level evaluation in patients with adrenal tumours. *Endocrine Regulations*.

[37] Takekoshi K, Isobe K, Yashiro T (2004). Expression of vascular endothelial growth factor (VEGF) and its cognate receptors in human pheochromocytomas. *Life Sciences*.

[38] Ohji H, Iciyanagi O, Suzuki Y, Nakada T, Sasagawa I (2001). Tumour angiogenesis and Ki-67 expression in phaeochromocytoma. *BJU International*.

[39] Chrisoulidou A, Kaltsas G, Ilias I, Grossman AB (2007). The diagnosis and management of malignant phaeochromocytoma and paraganglioma. *Endocrine-Related Cancer*.

[40] Ayala-Ramirez M, Chougnet CN, Habra MA (2012). Treatment with sunitinib for patients with progressive metastatic pheochromocytomas and sympathetic paragangliomas. *Journal of Clinical Endocrinology and Metabolism*.

